# Attraction Effect in Risky Choice Can Be Explained by Subjective Distance Between Choice Alternatives

**DOI:** 10.1038/s41598-017-06968-5

**Published:** 2017-08-21

**Authors:** Peter N. C. Mohr, Hauke R. Heekeren, Jörg Rieskamp

**Affiliations:** 1 Neuroeconomics, School of Business and Economics, Freie Universität Berlin, 14195 Berlin, Germany; 2 Neuroeconomics, Markets and Choice, WZB Berlin Social Science Center, 10785 Berlin, Germany; 3 Center for Cognitive Neuroscience Berlin (CCNB), Freie Universität Berlin, 14195 Berlin, Germany; 4 Biological Psychology and Cognitive Neuroscience, Department of Education and Psychology, Freie Universität Berlin, 14195 Berlin, Germany; 5 Center for Economic Psychology, Department of Psychology, University of Basel, 4055 Basel, Switzerland

## Abstract

Individuals make decisions under risk throughout daily life. Standard models of economic decision making typically assume that people evaluate choice options independently. There is, however, substantial evidence showing that this independence assumption is frequently violated in decision making without risk. The present study extends these findings to the domain of decision making under risk. To explain the independence violations, we adapted a sequential sampling model, namely Multialternative Decision Field Theory (MDFT), to decision making under risk and showed how this model can account for the observed preference shifts. MDFT not only better predicts choices compared with the standard Expected Utility Theory, but it also explains individual differences in the size of the observed context effect. Evidence in favor of the chosen option, as predicted by MDFT, was positively correlated with brain activity in the medial orbitofrontal cortex (mOFC) and negatively correlated with brain activity in the anterior insula (aINS). From a neuroscience perspective, the results of the present study show that specific brain regions, such as the mOFC and aINS, not only code the value or risk of a single choice option but also code the evidence in favor of the best option compared with other available choice options.

## Introduction

The assumption that decision makers evaluate choice options independently of each other is a cornerstone of standard economic choice theories. Standard theories postulate that the subjective value (or the expected utility) of an option does not change when another option is added to the choice set. Imagine a choice between two investments for retirement savings, an investment that offers a high return but also has a relatively high risk of low performance and another investment that offers a low return but is almost riskless. If you evaluate each of the two investments independently of each other, the addition of a third investment (e.g., medium return and risk) should not influence your relative preference between the original two investments. There is, however, a substantial amount of evidence showing that this independence assumption is frequently violated^1^. The choice set, i.e., the context in which an option is presented, can influence the evaluation of the option. Such context effects include the attraction effect (AE), the compromise effect (CE), and the similarity effect (SE). All three effects occur when a specific third option is added to a choice set, with a dominated option in case of the AE, an intermediate option in case of the CE, and a similar option for the SE. All three effects lead to a change of the relative preference for two choice options resulting from the addition of a third option. Previous studies examining context effects primarily focused on multi-attribute decision making without risk, such as consumer behavior. Only very few studies have so far investigated context effects in decision making under risk. These studies provide evidence that the AE can also occur for risky decisions for decisions alternatives with both discrete^2–4^ and continuous^5^ outcome distributions^2, 3^.

The change of relative preferences represents a violation of essential choice principles, i.e., the independence from irrelevant alternatives and the regularity principle, principles on which standard economic choice theories were built. Consequently, standard economic theories, such as the Logit or Probit model^6^, cannot explain context effects. The results of previous studies have led to the development of new choice theories explaining some of the violations^7^. However, new models that can, in principle, simultaneously explain all three context effects have also been developed, including Multialternative Decision Field Theory^8^ (MDFT), the Leaky, Competing Accumulator Model^9^ (LCA), the Associative Accumulation Model (AAM), and the Multi-Attribute Linear Ballistic Accumulator Model^10^. Notably, all four models belong to the class of sequential sampling models, which assume that a preference state develops over time by accumulating evidence for the different choice options^11^. All models further assume that the relationship of the choice options to each other within the multi-attribute space affects the subjective value of the options and is essential for explaining the context effects. MDFT, for instance, computes the psychological distance between options measured in two dimensions, the indifference and the dominance dimension. The indifference dimension defines a dimension within the multi-attribute space along which all options are equally attractive to the decision maker. The dominance dimension, in contrast, defines a dimension along which options dominate other options and therefore have a strict preference ordering. The overall distance is determined by an additive combination of the two distances where the importance weight given to each dimension strongly influences the effect on how options influence each other. The process in MDFT resulting in the AE is based on distance-based inhibition. The closer two options are with respect to each other in the space of relevant dimensions, the more these options are assumed to inhibit each other. If one of the two options dominates the other option, then the inhibition process results in a boost for the dominating option, thereby inverting the inhibition. This boost increases with decreasing distance. While distance-based inhibition is crucial in MDFT to account for the AE, the other three models mentioned above propose different processes. Whereas according to LCA, loss aversion in the value function leads to the AE, within the framework of the AAM a saliency-driven increase in the weight of the target’s strongest attribute is responsible for the AE. Finally, in MLBA alternatives that are more difficult to discriminate receive more attention, thereby increasing the choice probability of a dominant choice option in case of the AE. While all four models have been specified for multi-attribute decision making, it remains unclear how they should be applied to potential context effects in the domain of decision making under risk.

In the following we will focus on MDFT as a prominent representative of the sequential sampling models described. MDFT is an extension of decision field theory (DFT) that was explicitly developed to account for risky choices^12^. MDFT (i.e. “multi-alternative DFT”) is a generalization of DFT that can also be applied to choices between more than two alternatives. Furthermore, it includes mechanisms that allow MDFT to predict context effects. Thus, DFT cannot be applied to the three alternative choice tasks we study and we need to rely on MDFT. However, to allow MDFT to predict context effects for decision between risky alternatives, we represent probabilities and outcomes as two attributes (with logarithmized values). This procedure has already been successfully proposed by Wedell^4^. Taking the logarithm of both probability and outcome translates the two into additive attributes on which MDFT relies. However, this procedure nevertheless guarantees that the expected value model represents a special case of MDFT.

Only two studies so far have investigated the neural underpinnings of a context effect^13, 14^. The authors identified the AE in choices between flats and gambles. On the neural level, the authors of these studies observed higher brain activity in the dorsolateral prefrontal cortex (DLPFC), the anterior insula (aINS), and the dorsomedial prefrontal cortex (DMPFC) when a third asymmetrically dominated decoy was present, whereas brain activity in the amygdala and the ventromedial prefrontal cortex (VMPFC) was reduced in this situation. Both studies, however, did not provide a quantitative model to explain the effects and did not account for possible confounds of the number of presented alternatives (2 vs. 3 alternatives). Only recently have researchers started to explicitly model the data of context effects using quantitative models, such as MDFT^15^.

In the present study, we go beyond these past approaches by (a) providing further evidence that decisions under risk can be influenced by context effects, such as the AE, (b) examining whether these effects can be described by evidence accumulation models like MDFT, and (c) determining how the neurobiological underpinnings of the AE are related to the decision process assumed by MDFT.

MDFT assumes that similar choice options compete with each other and have a negative effect on each other. However, in the case of an unattractive dominated option with a negative overall evaluation, the competition leads to a positive “boosting” effect for the dominant option, as illustrated with the AE. How similar a dominated option is perceived according to MDFT depends on how much weight is given to the distances along the indifference compared with the dominance dimension. When giving little weight to the dominance dimension, the dominated option will be perceived as very similar to the dominant option, producing a large AE. Here, we specifically test how the overweighting parameter that determines the size of the AE is associated with changes in brain activity. In our study, we use fMRI to explicitly investigate how individual differences in the overweighting parameter are related to individual differences in decision-related brain activity.

**Figure 1:**
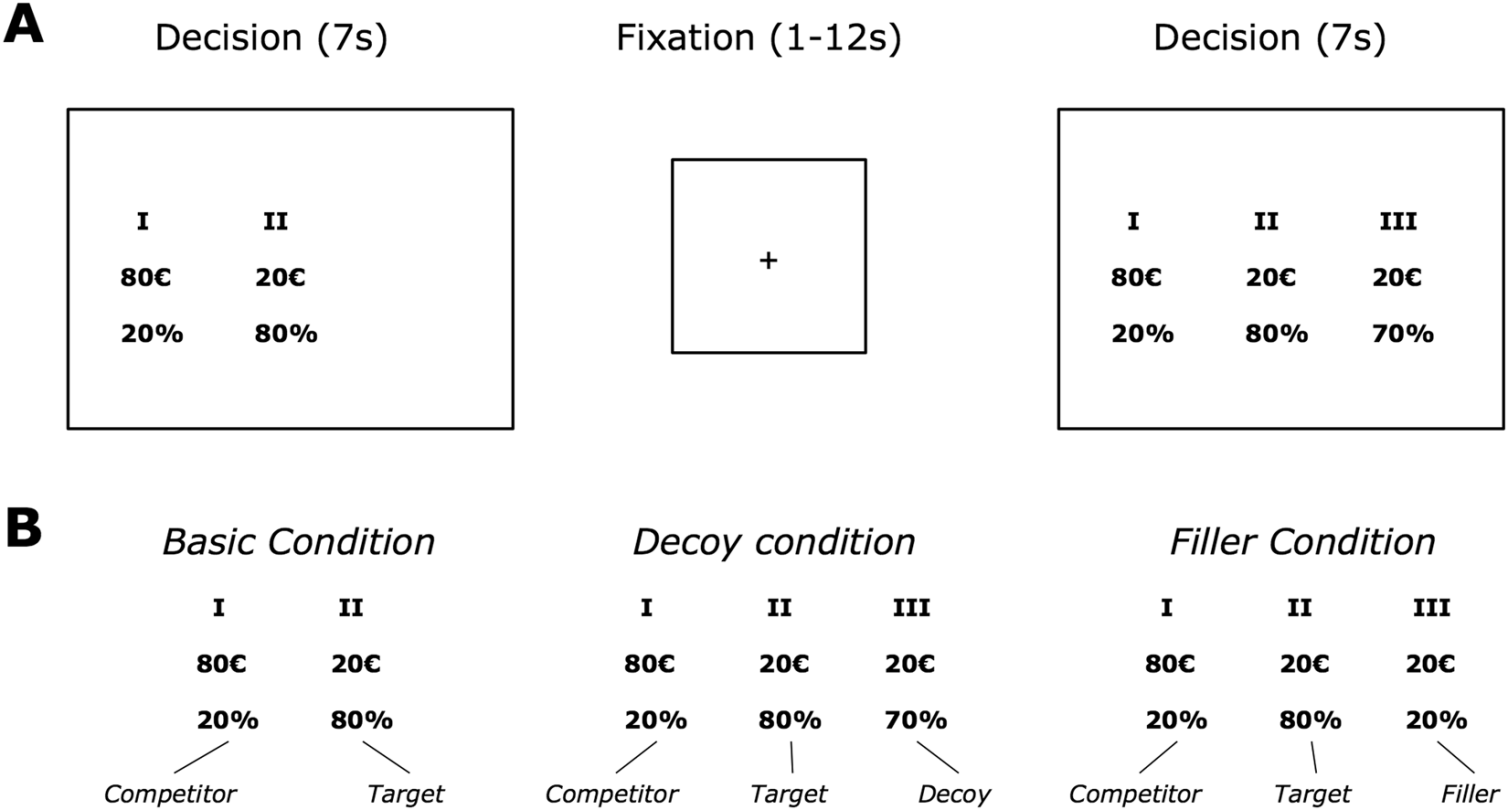
Task. (**A**) Subjects performed a series of choices between two or three risky gain-versus-nothing gambles and had 7 s to make their choice. (**B**) Choices were separated in three with-subject conditions, (I) the Baseline Condition, (II) the Decoy Condition, and (III) the Filler Condition. In (I) the Baseline Condition subjects made choices between two all-or-nothing gambles, one with a high probability to win a small amount of money (Target) and one with a low probability to win a high amount of money (Competitor). In (II) the Decoy Condition one option was added to the choice set compared to the Baseline condition. This additional option (Decoy) was similar to the option with a high probability to win a small amount of money, but the probability was exactly 10 percentage points lower to win the same small amount of money. In the (III) Filler Condition also one option (Filler) was added to the choice sets of the Basic Condition. The option, however, offered the same small win that was offered in one option but only with the low probability of the other option.

In our experiment, the participants (n = 18) made repeated choices between two or three monetary gambles during fMRI (see Fig. 1). The choice situations differed in three within-subject conditions: (I) choices between two options defined as the target and competitor option (basic condition), (II) choices between the target, the competitor, and a third option defined as the decoy, which was dominated only by the target but not by the competitor (decoy condition), and (III) choices between the target, the competitor, and a third dominated option symmetrically dominated by both the target and the competitor (filler condition). Importantly, according to a study-based preference task conducted prior to the main study, participants had to provide information concerning their indifference between different gambles, which provided us with information so that the target and competitor gambles were selected to make the participants indifferent between them (see the Methods section for details). Our analysis of the behavioral and neuroscientific data follows three steps. First, the different models are estimated on the basis of the choice data and rigorously compared by their goodness-of-fit (taking model complexity into account using the Akaike information criterion, AIC). Second, we test the ability of the winning model to account for the qualitative findings, that is the specific context effects we observed. Finally, we test whether the winning model can also be related to brain activity.

## Results

### Behavioral Data

**Figure 2:**
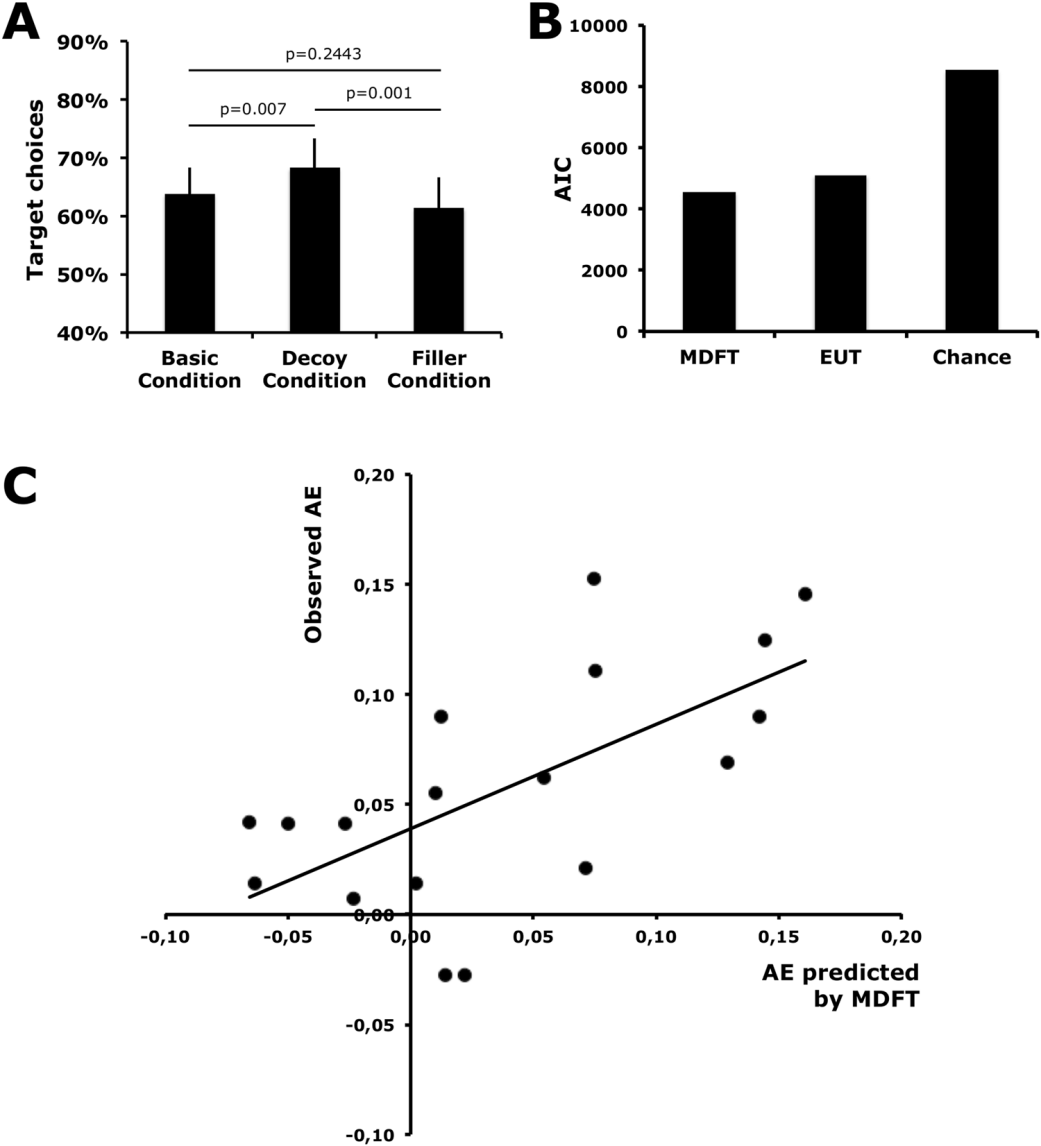
Behavioral Results. (**A**) Subjects chose the Target significantly more often in the Decoy Condition compared to the Baseline Condition or the Filler Condition. There was no significant difference in choice propensity between the Baseline and the Filler Condition. (**B**) In a model comparison, MDFT was better able to explain observed choices compared to EUT and a simple Chance Model, indicated by lower AIC scores. (**C**) Individual differences in the observed size of the AE were strongly correlated (r = 0.64) with individual differences in the size of the AE predicted by MDFT.

Paired t-tests of choice frequencies showed that the target was chosen significantly more often when a decoy was present compared with the other two conditions when no decoy was present (t = 3.097; p = 0.006) or a third symmetrically dominated option (t = 3.850; p = 0.001) was added to the choice set (see Fig. 2A). There were, however, no significant differences between the amount of target choices in the basic and the filler condition (t = 1.206; p = 0.244). This behavior represents a violation of the independence of irrelevant alternatives and the regularity principle and shows the manifestation of the AE in decision making under risk. We further applied MDFT to model participants’ choices. To examine whether MDFT described the data well, we rigorously tested it against Expected Utility (EU) theory and against a baseline chance model. According to the EU theory, the participants chose the option with the largest expected utility. Here, we defined the utility of the monetary outcomes using a power utility function with one free parameter (i.e., u(*x*) = *x* ^α^). The choice probability was specified using a softmax choice rule, including one sensitivity parameter. The baseline chance model assumed that all available choice options are chosen with equal probabilities. EU and MDFT must outperform the baseline model to represent plausible choice theories. The results showed that MDFT outperformed the other two models on an aggregated level (see Fig. 2B). On the individual level, MDFT outperformed the other two models for 16 out of 18 subjects according to the AIC^16^, which considers the complexity of the models. The EU theory performed best for two participants (see Figure S1 in the supplementary materials for details).

We also quantified the AE on an individual level by determining the difference in the choice frequency of the target option in the decoy condition compared with the average choice frequency of the target in the other two conditions, showing that the larger this difference, the larger the AE. To examine whether MDFT is able to predict the observed AE effect, we also computed the predicted AE effect by taking the difference between the average predicted choice probability of the target in the decoy condition compared with the average predicted choice probability of the target in the other two conditions. Figure 2C shows that the observed AE on an individual level was significantly correlated with the predicted AE by MDFT (*r* = 0.65; *p* = 0.004), with an average observed AE of 5.7% and a predicted effect of 3.8%. Thus, MDFT not only describes the choice data well but also accurately explains the AE on an individual level.

### fMRI Data

**Figure 3:**
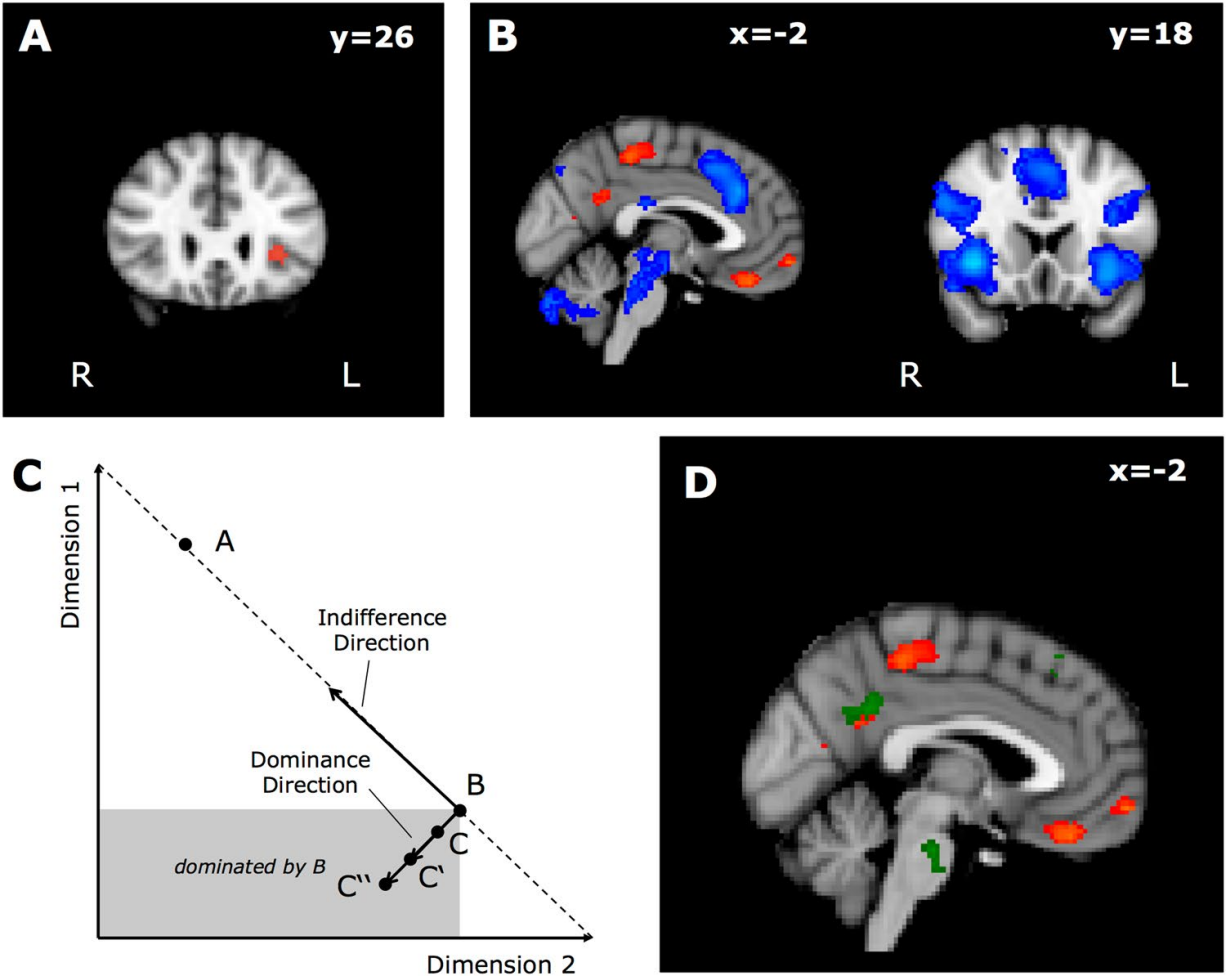
FMRI Results. (**A**) Brain activity in the aINS was significantly higher (z > 3.1, cluster size > 50, displayed in red) during decision making in the decoy condition compared to the filler condition. (**B**) Evidence in favor of the chosen option, operationalized as choice probability predicted by MDFT, showed a positive correlation (z > 3.1, cluster p < 0.05, displayed in red) with brain activity in mOFT/VMPFC and PCC and a negative correlation (z > 3.1, cluster p < 0.05, displayed in blue) with brain activity in bilateral aINS, bilateral DLPFC, and DMPFC. (**C**) In MDFT, the subjective distance between two alternatives is determined by the distance in the indifference direction and the distance in the dominance direction. Importantly, a specific parameter, namely the relative distance weighting parameter, allows for an overweighting of the distance in the dominance direction, leading to an increase in the subjective distance for dominated alternatives and a decrease in the AE. In the displayed example the subjective location of Option C would thus move to C’ or C” with an increasing relative distance weighting parameter. (**D**) Individual differences in the relative distance weighting parameter were related to neural representations of evidence in favor of the chosen option in PCC (z > 3.1, cluster p < 0.1, displayed in green). A decrease in the relative distance weighting led to decreased brain activity in the PCC.

As a first step in the fMRI analysis, we aimed at replicating the results of Hedgcock and Rao^13^, who observed higher brain activity in the DLPFC, aINS and DMPFC when an asymmetrically dominated decoy was present, whereas brain activity in the amygdala and the ventromedial prefrontal cortex (VMPFC) was reduced in this situation. We therefore contrasted constant brain activity during the decoy condition with constant brain activity during the baseline condition. Most of the observed differences indeed replicated the findings of Hedgcock and Rao^13^ (see Figure S2 in the supplementary materials for details). This contrast is, however, confounded with the comparison of choosing between three options (decoy condition) and choosing between two options (basic condition). Therefore, the observed differences might not specifically reflect the AE but rather the amount of processed information. We therefore contrasted constant brain activity in the decoy condition with constant brain activity in the filler condition, thereby holding the number of available options constant. Here, we identified only a relatively small cluster in the aINS (see Fig. 3A) that only appears to be significant when applying a very liberal cluster threshold (cluster size > 50) and fails to be significant for more rigorous thresholds (e.g., cluster-p < 0.1).

To relate brain activity to the MDFT, we examined whether the predicted choice probability of MDFT is correlated with changes in brain activity during choice. In general, the larger the predicted choice probability for the chosen option of MDFT the larger the accumulated evidence should be. In contrast, smaller choice probabilities indicate less evidence in favor of the chosen option and greater choice uncertainty. Consistent with this interpretation, we observed a positive correlation between the choice probability of the chosen option and brain activity in the medial orbitofrontal cortex (mOFC) and the posterior cingulate cortex (PCC) (see Fig. 3B). We further observed significant negative correlations between the choice probability and brain activity in the bilateral anterior insula (aINS), dorsomedial prefrontal cortex (DMPFC), thalamus, dorsolateral prefrontal cortex (DLPFC), and parietal cortex (see Fig. 3B). These regions have been identified in a meta analysis to code for risk in decision making^17^. Thus, these regions might not only code for uncertainty with respect to a specific choice option but also for decision uncertainty (see ref. 18, when accumulated evidence between options does not differ much. In general, the results indicate that the predictions of a sequential sampling model, such as MDFT, are to some extent consistent with the brain activity.

In a further step of the fMRI analysis, we specifically examined how the predicted AE of MDFT^19^ could be related to brain activity. As described above, the similarity between the options defined by the psychological distance between options is essential within MDFT for predicting the AE. According to MDFT, the subjective distance comprises the distance in the indifference dimension and the distance in the dominance dimension (see Fig. 3C). Importantly, the relative weighting of these distances influences how strongly the choice options inhibit or boost each other. The dominated option generally boosts the choice probability of the dominant option. The distance of the dominated option to the dominant option decreases with decreasing weight given to the dominance dimension, thus the underweighting of the dominance dimension should increase the AE. In a simulation we analyzed how underweighting of the dominance dimension relative to the indifference dimension increases the size of the AE. While maintaining all other estimated parameters constant on an individual basis, we parametrically varied the relative distance weighting parameter. We observed a positive correlation between underweighting of the dominance dimension and the AE size predicted by MDFT in all but 3 participants (mean r = 0.45, median r = 0.65). These results suggest that the weight given to the dominance dimension is indeed essential for MDFT to describe the AE. We therefore examined how the relative distance weighting parameter correlates with the brain activity.

We observed that the neural representation of evidence in the PCC is modulated by individual differences in the relative distance weighting parameter (see Fig. 3D). Crucially, subjects with lower overweighting of the distance in the dominance direction showed a decreased correlation between brain activity and predicted choice probability of the chosen option.

## Discussion

In the present study, we investigated (a) whether an individual’s decisions between risky options are influenced by the composition of the choice set, (b) whether these influences can be described using a sequential sampling model, such as MDFT, and (c) how the neurobiological underpinning of the AE could be related to the specific mechanisms assumed by MDFT to explain the AE.

The present results show that AE can also be observed in decision making under risk^2, 3^. Although the AE has previously been demonstrated in the literature^2, 20, 21^, these studies have primarily focused on consumer choices. More importantly, the effects have often been illustrated using between-subjects designs, leaving open whether the effects can also occur for the same person when making repeated decisions. To answer this question, we have used a within-subject design (see also ref. 15).

Several theoretical models have been proposed to account for the observed AE effects. One study showed that both the AE and the compromise effect increase when individuals have to subsequently justify their choices^22^. The authors argued that dominance or compromised relationships are used as tie-breaking reasons after a trade-off of the options’ attributes fails to lead to a clear preference. Another study compared three different classes of models that might account for the observed preference reversals in the AE^4^. In one class of models, the decoy is assumed to affect the weights assigned to different dimensions, whereas a second class of models proposes that the decoy produces range-frequency effects on dimensional values of alternatives. Finally, a third class of models, best able to explain the observed data, is, consistent with the ideas of Simonson^22^, based on the assumption that the perception of dominance directly increases the attractiveness of the dominating alternative. Another theoretical account focused on the weights individuals assign to the different attributes or dimensions^23^. The theory of dynamic choice reconstruction is based on the idea that the more similar two options are, the easier it is to recognize differences among their dimensions. This, in turn, would increase the relative weight of the dimension that differs changing also the preference relationship between two alternatives that are not similar.

Although the above described models account for the AE, few models to date can simultaneously explain all three context effects, namely the AE, the similarity effect, and the compromise effect^9, 10^. Here, we focused on MDFT^8^, which is based on the assumption that evidence is accumulated over time to form a preference state. By defining the logarithms of probability and outcome magnitude as inputs of MDFT, we established a model that includes expected value maximization as a special case. Similarly, EU theory also includes expected value maximization as a special case and thereby connects to MDFT. In a rigorous model comparison, we showed that MDFT explains observed choices better than EU theory and a baseline chance model. Additionally, we observed that the size of the AE is reflected in MDFT predictions. MDFT thus not only provides a theoretical account that explains the AE in general but also explains individual differences.

Only one study thus far investigated the neural underpinnings of the AE^13^. By comparing choices between two alternatives with choices between three alternatives (including a decoy) the authors showed that VMPFC and amygdala were less active when a decoy was present, whereas DLPFC, DMPFC, and aINS were more active. These authors attributed this finding to a general trade-off aversion causing the AE. Notably, however, the contrast between two alternatives with choices between three alternatives (including a decoy) is confounded with the mere amount of processed information. A comparison of all three conditions in the present study revealed that indeed most of the results of Hedgcock and Rao^13^ could be explained by comparing a choice between three options with a choice between two options, independent of the presence of a decoy and the AE. Importantly, in our experiment, only a small activation in the aINS remained when comparing the decoy condition with the filler condition and while holding the number of available options constant. As inferring cognitive processes from brain activity is in general problematic^24^, brain activity in the aINS is not selective, and the observed activation is only significant for very liberal cluster thresholds, we can only speculate on the functional role of the insula-activation for the occurrence of the AE. Changes in brain activity in the aINS have previously been attributed to saliency (ref. 25 for review), which was proposed to be an important driver of the AE in the AAM, a computational model similar to MDFT. Following the AAM, the AE is a result out of a saliency-driven overweighting of the target’s strongest attribute^26^. Future studies should address this potential interpretation of the insula-finding more explicitly.

As we were interested in how the neurobiological underpinnings of the AE could be related to the decision processes assumed by MDFT, we correlated choice probabilities predicted by MDFT with changes in brain activity during the decision. The predicted choice probability of the chosen option can be seen as a measure of accumulated evidence. A higher choice probability of the chosen option implies greater evidence in favor of the chosen option compared with the other options. We observed that evidence in favor of the chosen option is positively correlated with brain activity in mOFC/VMPFC and PCC and negatively correlated with brain activity in bilateral aINS, DLPFC, DMPFC, thalamus, and parietal cortex. Both the mOFC/VMPFC and the PCC have frequently been observed in the context of value processing^27, 28^. Other studies have associated the aINS, DLPFC, DMPFC, thalamus, and parietal cortex with the processing of risk^17, 29, 30^. The studies investigating value or risk processing, however, have focused either on the evaluation of a single object or on a choice between a varying alternative and a constant choice option. Thus, the question remains as to how the brain would code value and risk of multiple varying alternatives. The findings of the present study support the view that both networks might not only code for value and risk of single choice options but also for evidence in favor of the chosen option (e.g., in choices between two or more alternatives). Importantly, this interpretation is also consistent with the observations of previous studies investigating the value and/or risk of single choice options, as value and risk were fully confounded with evidence in favor of the chosen option in most tasks.

To investigate a possible connection between the choice process assumed by MDFT and brain activity during choices, we tested whether the relative distance weighting parameter of MDFT that is essential to predict the AE is associated with the neural processing of evidence. The process in MDFT resulting in the AE is based on distance-based inhibition. The closer two alternatives are with respect to each other in the space of relevant dimensions (in our case magnitude and probability of possible outcomes) the more these alternatives are assumed to inhibit each other. If one of the two options dominates the other option, then the inhibition process results in a boost for the dominating option, thereby inverting the inhibition. This boost is much stronger when the distance is smaller. An increase of the relative distance weighting parameter thus leads to a reduction of the boost and a decrease in the size of the AE. For high parameter values, the AE completely disappears, and the choices are similar to choices in choice sets without a decoy (e.g., in the basic condition of our experiment). We observed that individual differences in neural representations of evidence in the PCC are related to individual differences in the relative distance weighting parameter. Interestingly, the lower the relative distance weighting parameter (i.e., the higher the AE) the lower the relation between evidence and brain activity in the PCC became. The link between evidence (e.g., predicted by MDFT) and brain activity in the PCC is thus greatest when there is no AE present. Recent work by Grueschow and colleagues^31^ found that PCC reflects the subjective value of a choice alternative, even if the subjective value is irrelevant for choices (e.g., in perceptual decisions). As we interpret this activation in the context of multi-alternative choices as related to evidence, one potential interpretation of this finding would be that the PCC, in contrast to the mOFC/VMPFC, reflects unbiased evidence in favor of one option. This interpretation is in line with the findings by Grueschow and colleagues^31^. If the presence of a dominated option biases evidence in favor of one option, then this change results in a lower relation between brain activity in the PCC and evidence. The mOFC/VMPFC, in contrast, seems to be sensitive to the biasing effects of dominated options.

In summary, the present results show that in decision making under risk, the set of choice options influences the evaluation of single options, illustrated by the AE. MDFT provides a theoretical account that not only explains the AE in general but also explains individual differences in its size and brain activity related to the AE. From a neuroscience perspective, these results show that specific brain regions, such as mOFC/VMPFC, PCC or aINS, not only code value or risk of a single choice option but also provide evidence in favor of the best option when individuals must choose between several different options. The PCC thereby seems to code evidence in an unbiased way, not reflecting changes in evidence caused by the presence of a decoy option, whereas the mOFC/VMPFC fully reflect these changes.

Moreover, these results illustrate that the basic assumption of the independent evaluation of choice options embedded in standard economic choice theories can be substantially violated by human decision making. The results show that the value of choice options is affected by the context in which these options are presented, thus apparently, people do not simply reveal their preferences when making choices; on the contrary, they construct their preferences when making decisions in a specific situation. This idea suggests that, contrary to economic theory, the market price of an investment product might not only reveal the isolated preferences of the investors but also the impact of the context in which the investment product is presented.

## Methods

### Participants

Eighteen young volunteers (age 19–34 years, 7 females) participated in this study. All participants were native German speakers, right-handed, had no history of neurological or psychiatric diseases. All participants were paid for their participation and gave written informed consent. The study was approved by the local ethics committee of the Charité University Medicine, Berlin. All methods were performed in accordance with the relevant guidelines and regulations.

### Task

Participants performed a series of choices between gambles in three different conditions, (I) the Basic Condition, (II) the Decoy Condition, and (III) the Filler Condition. In (I) the Basic Condition participants made choices between two gambles, one with a high probability to win a small amount of money (e.g., 80%, 20$), and one with a low probability to win a high amount of money (e.g., 20%, 80$). Based on a pre-study preference task, these gambles were individually tailored in a way that the participants should be almost indifferent between the two options, ensuring that their choice behavior does not differ substantially from a 50%/50%-distribution. Specifically, we estimated two indifferences points that served as basis for stimulus generation. Participants initially made repeated choices between a choice option with 20% chance to win 80$ and an option with x% chance to win 20€. The x was varied parametrically in an increasing order. The x at which participants switched from choosing the first option to choosing the second option determines the indifference point. This procedure was repeated for choices between an option offering a chance of 25% to win 64$ and an option with a varying chance of y% to win 25€. The two indifference points were used to generate choice options for the main experiment. Here, we parametrically varied probability and magnitude in steps of 1%/1€ around the indifferences points.

In the Decoy Condition (II) one option was added to the choice set compared to the Basic Condition. This additional option, the decoy, was similar to the alternative with a high probability to win a small amount of money, but the probability was exactly 10 percentage points lower to win the same small amount of money (e.g., 70%, 20$). In the Filler Condition (III) also one option was added to the choice sets of the Basic Condition. The option offered the same small win that was offered in one option but only with the low probability of the other option (e.g., 20%, 20$). Participants made 72 choices in each condition. Additionally, we added 36 choices between a sure amount and a risky lottery to prevent participants from recognizing the aim of the experiment.

Participants received a flat payment of 10 Euro for their participation in the experiment. After the experiment, one of the 252 choices was randomly chosen to determine decision dependent payments. The option that was actually chosen on the randomly selected trial was played out, so that participants either won an additional amount of money in addition to their initial payment or just received no additional amount to their initial payment. The task was thus fully incentive compatible.

### Behavioral Modeling

We modeled behavioral data with three different models, namely a Baseline Chance Model, EU theory, and MDFT, and compared them in a rigorous model comparison. For the Model Comparison we used the AIC^16^ which penalizes additional parameters but is less conservative compared to the Bayesian Information Criterion (BIC). The Chance Model assumes equal choice probabilities for all available alternatives, that is ½ for two and 1/3 for three alternatives. For EU theory we assumed a simple power utility function of the form *u*(*x*) = *x* ^*q*^, where *u* defines the utility of outcome *x* and *q* the exponent is a free parameter that represents the risk attitude of the decision maker, with *q* = 1 representing risk neutrality, *q* < 1 risk aversion, and *q* > 1 risk seeking attitudes. Expected Utility (EU) was defined as the sum of the utilities of the outcome *x* multiplied with the probabilities *p* that the different outcomes *i* of an option occurred.

1 $$EU(m)=\sum _{i=1}^{I}{p}_{i}\cdot u({x}_{i})$$

To translate expected utilities into choice probabilities, we used a simple softmax choice rule:

2 $$Pr(m)=\frac{{e}^{\vartheta \cdot EU(m)}}{{\sum }_{j=1}^{N}{e}^{\vartheta \cdot EU(j)}}$$

where ϑ is a free sensitivity parameter and *N* denotes the number of available options of the choice set.

In MDFT, the evidence for each option at any point in time is captured by a preference vector *P*. *P* integrates all previous preference states *P* and the current evaluation *V* of the options according to the following updating process. The updating of *P* over time is assumed to continue until a specific threshold is reached, the decision time is over, or the preference states have stabilized. Here, we focused on the latter possibility. The preference state *P* is defined as:

3 $${P}_{t}=S{P}_{t-1}+{V}_{t},$$

where *S* describes a feedback matrix reflecting how strongly previous preference states are memorized (diagonal elements) and how the options influence each other, depending on their relative distance (*D*) in the attribute space and *V* representing the valence of the different options. The feedback matrix *S* is given by

4 $$S=\delta -{\phi }_{2}\cdot \exp (-{\phi }_{1}\cdot {D}^{2}),$$

where, δ is an identity matrix, φ_2_ a decay parameter that determines the diagonal elements of *S*, and φ_1_ a sensitivity parameter that determines the similarity as a function of the distance *D* between the options in the attribute space. The distance function *D* describes the subjective distance between two options in the attribute space. It is based on a decomposition of distance in the indifference direction and in the dominance direction. Importantly, a parameter *wd* allows for an overweighting of distance in the dominance direction and therefore an increase in subjective distance, if options are dominated.

MDFT assumes that options are evaluated relative to each other. This leads to dependencies in the evaluations of choice options that are reflected in the valence vector *V*_*t*_, which can be decomposed into three matrices and an error component (ε):

5 $${V}_{t}=CM{W}_{t}+\varepsilon ,$$

where, *C* denotes a contrast matrix to compute the relative advantage (disadvantage) of each option to the other options. *M* is a value matrix that contains the attribute values of each option and *W*_*t*_, represents a attention weight vector describing the relative importance of each attribute over time.

To estimate choice probabilities predicted by MDFT we assumed that individuals make choices after the preference states have stabilized and therefore set *t* to a very high level (*t* = 1000^15^). In the current form MDFT thus contains five free parameters, the weighting parameter *w*, the relative distance weighting parameter *wd*, the sensitivity parameter φ_1_, the decay parameter φ_2_, and a variance parameter *v* of the normally distributed error term ε. The probability and outcomes of a gamble are usually connected in a multiplicative way to determine, for instance, the expected value or the expected utility of an option. We therefore logarithmized the probabilities and outcomes in MDFT, so that it became sensible to use them as additive attributes and thereby expected value maximization becomes a special case of MDFT. We further follow the approach of Berkowitsch *et al*.^15^ to normalize attributes. Parameter estimation was performed in MATLAB R2015a (Mathworks), using the function fminsearch. We used the quantiles Q_0.25_, Q_0.5_, and Q_0.75_ of the estimated parameter distribution of Berkowitsch *et al*.^15^ as starting values within our optimization procedure. The combination of estimated parameter values resulting in the lowest AIC was used to determine the choice probabilities for every trial of the experiment to inform the fMRI analysis.

### MRI Data Acquisition

Whole-brain fMRI data were collected on a 3 T Siemens Magnetom Trio scanner using a 12-channel phased-array head coil. Echoplanar images (EPI) were acquired from 36 axial slices of 68 × 68 voxels with 3-mm in-plane resolution, 3.45-mm slice thickness, field of view (FOV) of 204 mm, 80° flip angle, 28-ms echo time (TE), and 2-s repetition time (TR). For each participant two runs of 670 volumes each were acquired. Initial scout scans were performed to localize slice planes parallel to the anterior commissure – posterior commissure line. In addition, high-resolution T1-weighted structural scans were collected for each participant for registration purposes using an MPRAGE sequence (192 sagittal slices; matrix size: 256 × 256; voxel size: 1 × 1 × 1 mm; FOV: 256 mm; flip angle: 9°; TE: 2.52 ms; TR: 1.9 s).

### MRI Data Analysis

MRI data were analyzed using a mixed effects approach within the framework of the general linear model as implemented in the FMRI Expert Analysis Tool (FEAT^32^, part of FSL 4.0 (FMRIB’s Software Library, http://fmrib.ox.ac.uk/fsl). Pre-processing included slice-timing correction, motion correction, and spatial smoothing using an 8 mm Gaussian kernel. Additionally, pre-whitening was used and high-pass temporal filtering (90 s) was applied to the data. A double-gamma function was used to model the hemodynamic response.

Event-related fMRI data were analyzed using General Linear Models (GLM) with task-related regressors. Images of individual level regression parameters (contrast images) were normalized into a standard stereotaxic space (Montreal Neurological Institute (MNI), Montreal, Quebec, Canada) and included a random-effects group analysis. Here we used the Bayesian modeling approach^33^ implemented in FSL’s FLAME (FMRIB’s local analysis of mixed effects) procedure to test whether regression parameters were significantly different zero, or significantly different from each other. For the a priori regions of interest we report those activations as significant that exceed an uncorrected threshold of *z-score* > 3.1 and a cluster size > 50. A priori regions of interest were regions that have been shown to be implicated in preferential decision making^34–36^. These regions include the prefrontal cortex, the cingulate cortex, the striatum, the aINS, the amygdala, the hippocampus, and the parietal cortex.

We analyzed two different models. The purpose of GLM1 was to replicate the findings of Hedgcock and Rao^13^. We therefore defined three regressors of interest and one regressor of no interest. The regressors of interest modeled constant brain activity during the decision phase (7 s) of the three conditions of our experiment (Basic Condition, Decoy Condition, and Filler Condition). Additionally, we included one regressor of no interest that modeled the decision phase (7 s) of missed trials as well as choices between a sure amount and a risky option that were included to prevent subjects from recognizing the aim of the experiment (see above). Each of the three regressors of interest was contrasted against the other two.

The purpose of GLM2 was to examine the relationship between evidence in favor of the chosen option and brain activity. Here, we included two regressors of interest and one regressor of no interest. One regressor of interest (R1) modeled brain activity during the decision phase (7 s) independent of the condition. The second regressor of interest (R2) modeled a parametric modulation of the decision phase (7 s) with the choice probability of chosen option that was predicted by MDFT. The regressor of no interest modeled the decision phase (7 s) of missed choices (if there were any). Importantly, R2 was orthogonalized with respect to R1. Here, we tested in which brain regions regression parameters of R2 are significantly different from zero (positive or negative). We further analyzed in which brain regions individual differences in the relative distance weighting parameter of MDFT are related to individual differences in regression parameters obtained from R2.

### Data Availability

The datasets generated and analyzed during the current study are available from the corresponding author on reasonable request.

## Electronic supplementary material

Supplementary Information
