# Supplementary material for: Attraction Effect in Risky Choice Can Be Explained by Subjective Distance Between Choice Alternatives
Source: Sci Rep. 2017 Aug 21;7:8942. doi: 10.1038/s41598-017-06968-5 (PMC5567099; doi:10.1038/s41598-017-06968-5)
Supplement: Supplementary file 1 — Supplementary Information [file 41598_2017_6968_MOESM1_ESM.pdf]

Attraction Effect in Risky Choice Can Be Explained by Subjective Distance Between Choice Alternatives

Peter N.C. Mohr[a,b,c,*], Hauke R. Heekeren[c,d], and Jörg Rieskamp[e]

[a]Neuroeconomics, School of Business and Economics, Freie Universität Berlin, 14195 Berlin, Germany
[b]Neuroeconomics, Markets and Choice, WZB Berlin Social Science Center, 10785 Berlin, Germany
[c]Center for Cognitive Neuroscience (CCNB), Freie Universität Berlin, 14195 Berlin, Germany
[d]Biological Psychology and Cognitive Neuroscience, Department of Education and Psychology, Freie Universität Berlin, 14195 Berlin, Germany
[e]Center for Economic Psychology, Department of Psychology, University of Basel, , 4055 Basel, Switzerland

[*]Corresponding author

Classification:
Biological sciences; Neuroscience; Reward

Supplementary Information

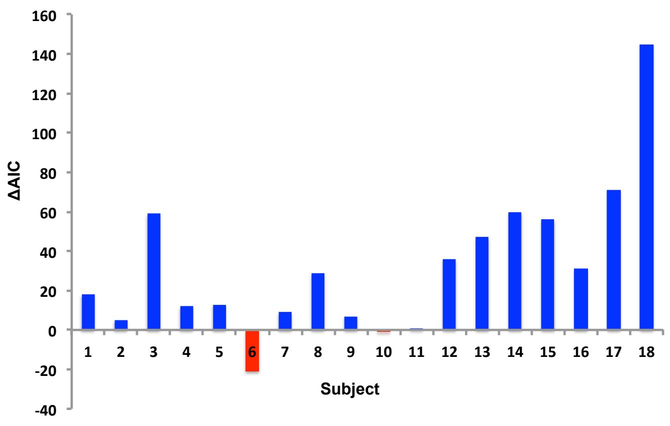

Figure S1. Relative model fit. Blue bars denote subjects with a lower AIC score for MDFT. Red bars describe subjects with a lower AIC score for EUT.

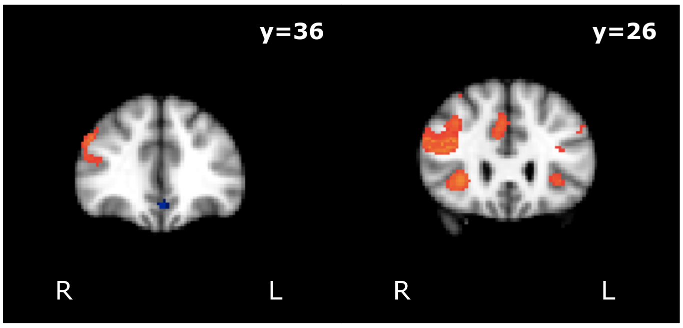

Figure S2. FMRI Results. Comparison between brain activity in the decoy condition and the basic condition (GLM1). Higher BOLD signal (z>3.1; cluster size > 50) in the decoy condition in DMPFC, DLPFC, and aINS (all displayed in red). Lower BOLD signal (z>3.1; cluster size > 50) in the decoy condition in mOFC/VMPFC (displayed in blue).
